# Cardiogenic shock: all hail the RCT, long live the registry

**DOI:** 10.1186/s13054-024-04835-0

**Published:** 2024-02-19

**Authors:** A. Warren, D. Morrow, Alastair G. Proudfoot

**Affiliations:** 1 Barts Heart Centre, St. Bartholomew’s Hospital, West Smithfield, London, EC1A 7BE UK; 2 Division of Cardiovascular Medicine, Brigham and Women’s Hospital, Boston, MA USA; 3 Harvard Medical School, Harvard University, Boston, MA USA; 4 Critical Care and Perioperative Medicine Group, Queen Mary University London, London, UK

**Keywords:** Cardiogenic shock, Registry, Randomised controlled trial, Observational data

The persistently and unacceptably high mortality of cardiogenic shock (CS) [1] demands the pursuit of evidence to confirm the efficacy and safety of existing and novel interventions at a population level. Randomised controlled trials (RCTs) remain the cornerstone of evidence generation. Nonetheless, sobering neutral clinical trial results [2, 3] and either protracted [4] or failed [5] recruitment have brought into question whether we currently have the capabilities to effectively design and execute clinical trials in CS. Therefore, self-critical re-examination of our approach to advancing the care of cardiogenic shock is essential.

Serial neutral clinical trials in other fields of critical care have arguably tempered enthusiasm of both academics and industry to invest resources. Given the risk of sunk research costs, healthcare systems may also elect to target clinical activity at the expense of trial enrolment. Adaptive platform trial designs and a shift towards Bayesian methods coupled with the results from soon to report, or currently enrolling trials may buck this trend. Nonetheless, the cost and time required to execute RCTs are likely to remain substantial and the need for complementary, less costly and more efficient methods of evidence generation will persist. As such, the concomitant growth of high-quality research registries is essential to understand the clinical landscape of CS, enhance future clinical trial design and execution, and ensure that investment in CS research remains a priority.

The majority of prior CS trials have recruited only patients with acute myocardial infarction, which is the primary aetiology in less than half of patients [1]. Compared to observational studies, patients included in CS trials have been more often male, have had fewer co-morbidities, received less advanced therapies, and had higher mortality [6]. The research community has made recent advances through standardisation of definitions for future clinical trials and registries [7] but there remains considerable uncertainty regarding case selection for and optimal timing of trial interventions. Furthermore, there is a lack of consensus, let alone evidence, to inform what ‘standard care’ should be prescribed for patients in both the control and intervention arms of comparative effectiveness trials.

The immediate focus of cardiogenic shock registries should be to disentangle the heterogeneity of the syndrome that may account for the variability of treatment effect documented across trials and observational studies. There is an unmet need to characterise or phenotype patients who are most likely to benefit (or at lowest risk of harm) from trial interventions, specifically around mechanical circulatory support (MCS) which is associated with considerable complication rates. It is unlikely that simply increasing the sample size of future RCTs is feasible or will overcome the challenges of this heterogeneity [4].

To truly complement RCTs, registries should include all patients with the syndrome regardless of aetiology. Crucially, granular, longitudinal data describing shock aetiology, investigations and management including detailed haemodynamic data are essential to better describe standard care and to identify the optimal window for potential intervention or failure to respond to first-line treatments. Current exemplars of this approach are the Cardiac Critical Care Trials Network, Cardiogenic Shock Working Group and VANQUISH registries (Table 1). At present, these registries largely contain patients from tertiary academic hospitals and hence are unlikely to represent the complete landscape of CS and may reflect selection pressures similar to those seen in clinical trials. The American Heart Association and UK Intensive Care National Audit and Research Centre registries are collecting pragmatic data at a national level targeting both research and quality improvement outputs. The value of a national approach to data collection and quality improvement has been realised in Denmark where the National Cardiac Arrest Registry has significantly improved rates of bystander cardiopulmonary resuscitation, public-access defibrillator use and survival in out-of-hospital cardiac arrest [8].

**Table 1:** Examples of currently active multicentre research registries enrolling patients with cardiogenic shock

Name	Location	Start date	Centre n	Inclusions
*Disease-based registries*				
Cardiac Critical Care Trials Network (CCCTN)	US, Canada, UK	2019	42	All CS aetiologies, high volume CICUs
VANQUISH	US, Canada	2022	4	All CS aetiologies, high-volume CICUs
ICNARC Cardiogenic Shock Registry**	UK	2023	6**	All CS aetiologies, all hospital types
Altshock-2 registry (https://doi.org/10.1002/ccd.30484)	Italy	2020	11	All CS aetiologies, advanced MCS centres
Cardiac Shock Working Group (CSWG)	US	2016	15	All CS aetiologies, high-volume CICUs
AHA Cardiogenic Shock Registry	US	2022	54	All CS aetiologies, all hospital types
*Device-based registries*				
Extracorporeal Life Support Organisation (ELSO)	International	1990	634^§^	Patients receiving “cardiac” ECMO
Japan Percutaneous Left Ventricular Assist Device Registry (J-PVAD)	Japan	2017	109	Patients receiving Impella ®
National Cardiogenic Shock Initiative (NCSI)	US	2016	80	Patients with AMI-related CS receiving Impella®

*CICU* cardiac intensive care unit; *VANQUISH* The Multicenter Collaborative to Enhance Biological Under- standing, Quality and Outcomes in Cardiogenic Shock VANQUISH Shock; *ICNARC* Intensive Care National Audit and Research Centre; *AHA* American Heart Association; *MCS* Mechanical Circulatory support

**Personal correspondence, In pilot phase. Anticipated centre size of 150–200

^§^As per the “adult cardiac” subgroup from ELSO report 2023

Other registries collect data only on CS patients supported with MCS devices (Table 1), such as the Extracorporeal Life Support Organisation (ELSO) registry for patients receiving extracorporeal membrane oxygenation [9] and several registries for patients supported with the Impella® microaxial pump [10, 11]. While providing useful insights, these registries were not designed to collect data on the full spectrum of cardiogenic shock. There is also significant confounding by indication in descriptive comparative conclusions drawn from device registries without corresponding data from patients who were not eligible, not referred, or not accepted for device therapy.

It is important that registries are adequately resourced to support longitudinal data collection, rather than time-restricted data. Several groups have executed large-scale multicentre observational cohort studies, including FRENSHOCK [12] (France), SMART RESCUE [13] (South Korea), and the JCS Cardiovascular Shock Registry (Japan) [14]. While commendable, these efforts included patients only within a given timeframe. Hence, secular trends that may impact RCT inclusion or design may be missed. For example, the DanShock study extended recruitment into Germany after lower than anticipated rates of more severe CS meeting the trial inclusion criteria meant less than a third of the target 360 patients had been recruited over 6 years of study [4].

The implementation of registries and the interpretation of observational data are not without challenges; confounding and selection bias; data quality; the resource required to efficiently collect, curate, securely store and maintain data; the requisite ethical and data governance approvals. These obstacles aside, in the face of repeated neutral clinical trial results, there is a compelling argument for continued and renewed investment in CS registries to engineer opportunities for quality improvement and to complement and inform future RCT design. Exploration and, crucially, delineation of the heterogeneity of the CS syndrome and its treatment responses is essential to for the design of future RCTs. Registry data is also crucial to guide site recruitment and enrolment planning. Finally, registries offer the opportunity to embed RCTs within their infrastructure, a concept that is outlined eloquently elsewhere [15].

To improve outcomes in CS, we need to target the right patients, at the right time in their disease trajectory with the interventions most likely to improve outcome and that can be applied at a population level. Registries remain fundamental in this endeavour.

## Data Availability

Not applicable.

## Abbreviations

MCS: Mechanical circulatory support
RCT: Randomised controlled trial
CS: Cardiogenic shock
